# Navigating a Dual Hyperinflammatory State: Overlap of Drug Reaction With Eosinophilia and Systemic Symptoms (DRESS) and Secondary Hemophagocytic Lymphohistiocytosis (HLH) in a Child

**DOI:** 10.7759/cureus.100848

**Published:** 2026-01-05

**Authors:** Aashi Gupta, Bimlesh Prasad, Ajit Dhumale, Kundavaram Rajkumar, Vikrant S Bhar, Shruti Gupta

**Affiliations:** 1 Pediatrics, Dr. Ram Manohar Lohia Institute of Medical Sciences, Lucknow, IND; 2 Pediatrics, All India Institute of Medical Sciences, Raebareli, Raebareli, IND; 3 Pathology, All India Institute of Medical Sciences, Raebareli, Raebareli, IND

**Keywords:** dress, hlh, hyperinflammatory state, phenytoin, phenytoin dress secondary hlh hyperinflammatory state, secondary

## Abstract

Drug reaction with eosinophilia and systemic symptoms (DRESS) is a severe drug-induced hypersensitivity reaction with a diverse clinical presentation. Hemophagocytic lymphohistiocytosis (HLH) is a life-threatening hyperinflammatory syndrome due to uncontrolled immune activation. Although uncommon, overlap between DRESS and secondary HLH is increasingly reported, particularly in association with viral reactivation such as Epstein-Barr virus (EBV). Both DRESS and HLH can present with multi-organ dysfunction. We present a 12-year-old boy who presented with fever for four weeks and a generalized maculopapular rash for three weeks after the initiation of phenytoin, and a diagnosis of DRESS was made after skin biopsy. Withdrawal of phenytoin and initiation of systemic corticosteroids led to initial clinical improvement, but the clinical course was complicated by secondary HLH due to EBV reactivation. Treatment with dexamethasone and etoposide resulted in recovery of clinical symptoms and normalisation of biochemical parameters. This case underscores the importance of early recognition of HLH in children with DRESS who show clinical deterioration.

## Introduction

Drug reaction with eosinophilia and systemic symptoms (DRESS), also known as drug-induced hypersensitivity syndrome (DIHS), is a rare but serious delayed drug reaction that typically appears two to eight weeks after initiating high-risk medications, such as phenytoin, carbamazepine, sulfonamides, or allopurinol [1]. Characteristic features include a widespread rash (often morbilliform and occasionally exfoliative), facial edema, persistent fever, generalized lymphadenopathy, eosinophilia, and internal organ involvement, most commonly affecting the liver or kidneys. Although uncommon, DRESS carries a substantial risk of complications and has a mortality rate of up to 10% [2]. The criteria proposed by the International Registry of Severe Cutaneous Adverse Reactions (RegiSCAR) group involve clinical and laboratory findings in establishing the diagnosis as a possible case (scores 2-3), probable case (scores 4-5), and definitive case of DRESS (scores more than 5).

Hemophagocytic lymphohistiocytosis (HLH) is a life-threatening hyperinflammatory syndrome resulting from uncontrolled immune activation. It may be inherited or secondary to infections, malignancies, autoimmune diseases, or drug reactions. The overlap between DRESS and HLH, though rare, is increasingly recognized, with viral reactivation - particularly Epstein-Barr virus (EBV) - playing a central role in this progression [3]. We report a paediatric case of phenytoin-induced DRESS complicated by secondary HLH triggered by EBV reactivation, underscoring the importance of early recognition and timely intervention in such complex immune-mediated disorders [4].

## Case presentation

A 12-year-old boy presented with high-grade fever of four weeks' duration. He also developed a generalized blanchable maculopapular rash that began on the trunk on day five of fever and later spread to the limbs. His past history was significant for head trauma following a road traffic accident, after which he had been receiving phenytoin for three months. On examination, he had facial puffiness, generalized edema, splenomegaly, and tender lymphadenopathy involving the cervical, axillary, and inguinal regions. He was evaluated for infectious causes, started on broad-spectrum antibiotics, and investigated for organ dysfunction. Initial laboratory findings revealed leukocytosis, eosinophilia, atypical lymphocytes on peripheral smear, and deranged liver function tests. The laboratory parameters are summarized in Table 1.

**Table 1:** Laboratory work up in our patient

Parameter (Units)	Patient value	Reference range
Hemoglobin (gm/dL)	0.8	11.5-16
Total leucocyte count (10³/μL)	32.1	4-11
Platelet (10³/μL)	296	150-450
C-reactive protein (mg/dL)	0.91	Less than 6
Erythrocyte sedimentation rate (mm/hour)	20	0-15
Total bilirubin (mg/dL)	8.64	0.2-1.2
Direct bilirubin (mg/dL)	5.65	0.1-0.5
Indirect bilirubin (mg/dL)	2.99	0.2-1.2
Serum glutamic oxaloacetic transaminase (U/L)	2192.6	5-45
Serum glutamic pyruvate transaminase (U/L)	1693.3	54-369
Serum urea (mg/dL)	22.4	10-45
Serum creatinine (mg/dL)	0.39	0.6-1.6

**Figure 1:**
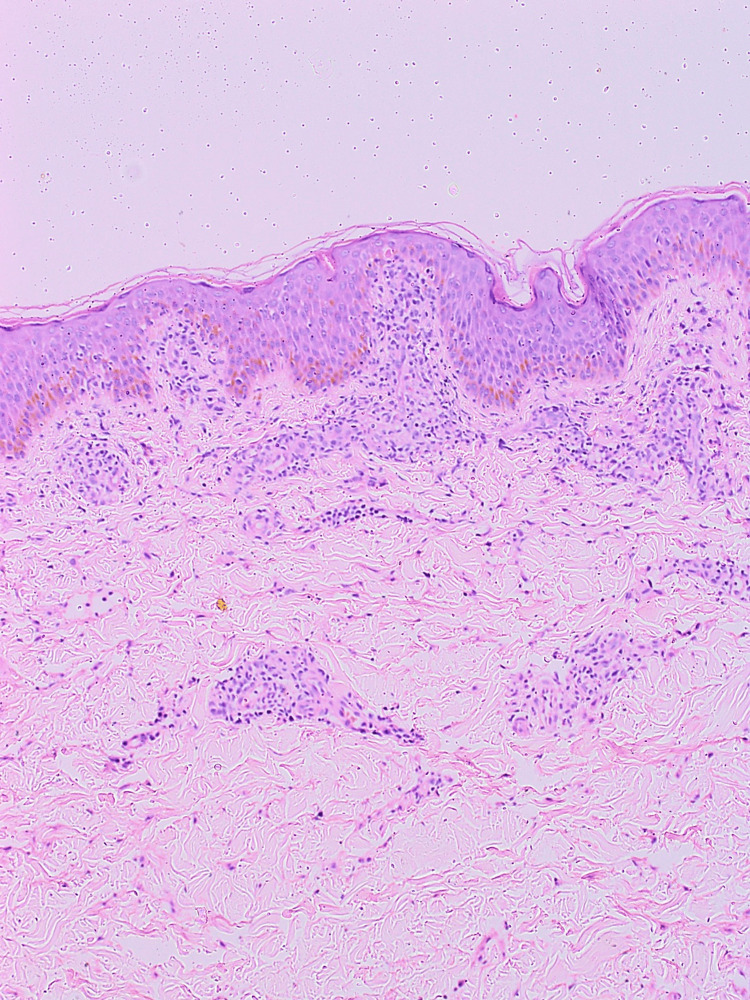
Skin punch biopsy The microphotograph shows unremarkable epidermis. Superficial dermis shows a moderate amount of lymphoplasmacytic infiltrates and scattered histiocytes.

Given the endemic prevalence, scrub typhus was considered; scrub PCR was sent, and azithromycin was initiated. However, the child showed no clinical improvement. Further evaluation included fine-needle aspiration of a lymph node, which revealed reactive lymphoid hyperplasia with activated lymphocytes. A skin punch biopsy showed moderate lymphoplasmacytic infiltrates and scattered histiocytes in the dermis (Figure 1).

**Figure 2:**
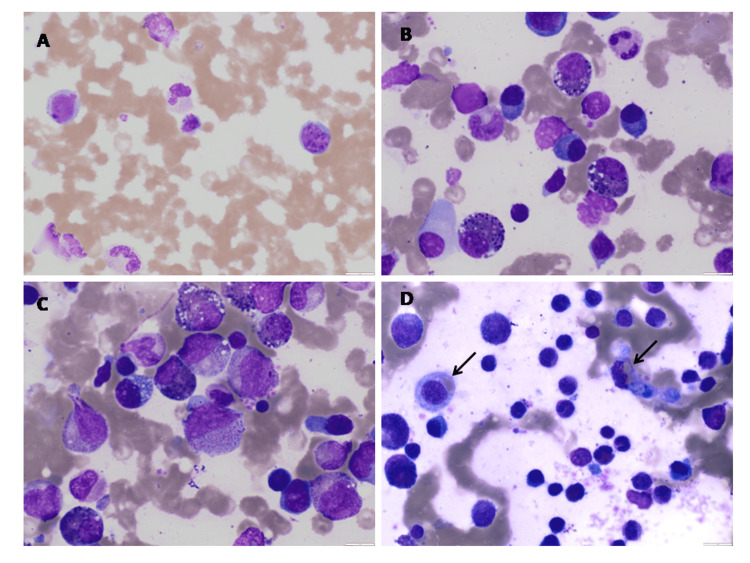
Peripheral smear, bone marrow aspiration, and biopsy A. Peripheral smear shows activated lymphocytes and degenerated lymphocytes undergoing nuclear fragmentation. B. Bone marrow aspiration smear increased plasma cells and many eosinophilic myelocytes with vacuolations. C. Bone marrow aspiration smear shows prominent granulations of neutrophilic lineage, increased eosinophilic precursors and cytoplasmic vacuolations. D. Macrophages (black arrow) showing erythrophagocytosis in the bone marrow.

The constellation of fever, rash, lymphadenopathy, eosinophilia, atypical lymphocytes, and multi-organ involvement (liver and kidneys) raised suspicion for DRESS. On detailed review, the onset of fever and rash roughly eight weeks after initiating phenytoin further supported this diagnosis. The RegiSCAR score was 7, consistent with definite DRESS. Phenytoin was discontinued, and the child was started on 2 mg/kg/day of oral prednisolone. Initially, the child improved with resolution of fever and rash and normalization of laboratory parameters. However, fever persisted, splenomegaly with worsening transaminases. Serum ferritin rose dramatically to 4,000 ng/mL. Given the features and markedly elevated inflammatory markers, HLH was suspected. Serum triglycerides were elevated at 281.6 mg/dL and hypofibrinogenemia (136 mg/dL), indicating intense immune activation. Bone marrow aspiration and biopsy demonstrated hemophagocytosis (Figure 2).

Five out of eight HLH parameters were seen in our patient, fulfilling the diagnostic criteria for HLH (laboratory details in Table 2).

**Table 2:** HLH parameters seen in our patient HLH: Hemophagocytic lymphohistiocytosis

S No	HLH Parameter
1	Fever (temperature 104.6 F)
2	Splenomegaly (5 cm)
3	Hypertriglyceridemia (281.6 mg/dL) (reference ≥ 265 mg/dL), hypofibrinogenemia (136 mg/dL) (reference ≤ 1.5g/L)
4	Hemophagocytes on bone marrow (Yes)
5	Hyperferritinemia (4000) (reference ≥ 500 µg/L)

We hypothesized that secondary HLH was triggered by EBV reactivation. This was confirmed by an EBV DNA load of 1.8 × 10³ copies/mL, with positive serum IgM and IgG for EBV viral capsid antigen - suggesting acute reactivation on a background of past exposure. Whole-exome sequencing was normal, excluding primary HLH. The temporal sequence - initial DRESS followed by acute deterioration with laboratory evidence of EBV reactivation - supported a diagnosis of secondary HLH precipitated by EBV in the setting of DRESS. The child was treated with intravenous dexamethasone, with dramatic clinical and biochemical improvement. He was discharged in stable condition and, at three-month follow-up, had normal laboratory parameters and had resumed school.

## Discussion

Although DRESS is more commonly recognized in adults, paediatric cases - though less frequent - may present with more severe systemic involvement. Several factors contribute to DRESS, including genetic susceptibility, abnormal drug metabolism, and immune dysregulation [5]. Diagnosis in children requires a high index of suspicion, as the presentation often mimics infections or autoimmune disorders, and symptoms typically develop two to eight weeks after drug exposure. In our patient, the diagnosis of DRESS was considered only during the second week of admission when he failed to respond to antibiotics and infectious evaluations returned negative.

A hallmark of DRESS is the reactivation of latent viruses, such as HHV-6, EBV, or CMV, which contributes to immune dysregulation [5]. When HLH develops in the context of DRESS, it represents a rare but increasingly recognized overlap syndrome. EBV reactivation, in particular, may amplify the immune response, predisposing to HLH [6]. Elevated cytokines - including IL-5, IL-6, IL-10, and soluble IL-2 receptor - are seen in both DRESS and HLH and contribute to the severe inflammatory state [7].

Management of DRESS includes immediate withdrawal of the offending drug and supportive care [8]. Systemic corticosteroids are the mainstay for moderate to severe cases and are generally effective [9]. In children, intravenous steroids often lead to rapid improvement. For severe multi-organ involvement or myocarditis, intravenous immunoglobulin (IVIG) may also be considered [10].

In our patient, drug-induced immune activation and EBV reactivation likely triggered the transition from DRESS to HLH, a pattern similar to that reported in the literature. Early recognition of HLH in DRESS is crucial [6]. Warning signs include persistent or relapsing fever, cytopenias, and worsening organ dysfunction. Although some laboratory abnormalities overlap, features such as coagulopathy and hemophagocytosis more strongly suggest HLH. Evaluation for HLH is recommended in DRESS patients with unusually severe, prolonged, or relapsing illness [8]. Bone marrow examination, though not always essential for diagnosis, can confirm hemophagocytosis and exclude malignancy [9]. Treatment typically includes dexamethasone with or without etoposide, as used in our patient.

Long-term follow-up is essential, as patients recovering from DRESS may later develop autoimmune complications such as thyroiditis or lupus-like illness. Those treated with cytotoxic agents such as etoposide also require surveillance for late effects, including secondary malignancies and reproductive health concerns [9]. The prognosis of DRESS-associated HLH is guarded, with mortality reported as high as 30% in some series, with paediatric deaths documented in cases triggered by drugs such as minocycline and allopurinol [10]. Our patient’s favourable outcome was likely due to early identification and prompt treatment.

## Conclusions

A high index of suspicion is essential for early recognition of DRESS and timely discontinuation of the offending drug. Viral reactivation is common in DRESS and may precipitate secondary HLH. Aggressive immunotherapy is key along with blood products transfusion. Multidisciplinary management and long-term follow-up are crucial. Families should be counselled on avoidance of offending to prevent recurrence.
